# Performance improvement of phase-change memory cell using AlSb_3_Te and atomic layer deposition TiO_2_ buffer layer

**DOI:** 10.1186/1556-276X-8-77

**Published:** 2013-02-15

**Authors:** Sannian Song, Zhitang Song, Cheng Peng, Lina Gao, Yifeng Gu, Zhonghua Zhang, Yegang Lv, Dongning Yao, Liangcai Wu, Bo Liu

**Affiliations:** 1 State Key Laboratory of Functional Materials for Informatics, Shanghai Institute of Micro-system and Information Technology, Chinese Academy of Sciences, 865 Changning Road, Shanghai, 200050, China; 2 Division of Nuclear Materials Science and Engineering, Shanghai Institute of Applied Physics, Chinese Academy of Sciences, 2019 Jialuo Road, Jiading District, Shanghai, 201800, China; 3 National Laboratory for Infrared Physics, Shanghai Institute of Technical Physics, Chinese Academy of Sciences, Shanghai, 200083, China

**Keywords:** phase change memory, atomic layer deposition, TiO_2_ buffer layer, reset voltage, AlSb_3_Te

## Abstract

A phase change memory (PCM) cell with atomic layer deposition titanium dioxide bottom heating layer is investigated. The crystalline titanium dioxide heating layer promotes the temperature rise in the AlSb_3_Te layer which causes the reduction in the reset voltage compared to a conventional phase change memory cell. The improvement in thermal efficiency of the PCM cell mainly originates from the low thermal conductivity of the crystalline titanium dioxide material. Among the various thicknesses of the TiO_2_ buffer layer, 4 nm was the most appropriate thickness that maximized the improvement with negligible sacrifice of the other device performances, such as the reset/set resistance ratio, voltage window, and endurance.

## Background

Phase change memory (PCM) has been regarded as the one of the most promising nonvolatile memories for the next generation because of the advantages of high speed, low power, good endurance, high scalability, and fabrication compatibility with complementary metal-oxide-semiconductor (CMOS) process [1-4]. PCM uses the reversible phase change between the crystalline and amorphous states of chalcogenide materials brought about by Joule heating. Ge_2_Sb_2_Te_5_ (GST) is the most widely used due to its relatively good trade-off between thermal stability and crystallization speed. However, with low crystallization temperature (around 140°C), GST is susceptible to the issue of thermal cross-talk by the proximity effect [5]. The high reset current (mA) results in high power consumption for GST-based PCM [6]. The switching speed, which is limited by its nucleation-dominated crystallization mechanism, is insufficient to satisfy the requirement of dynamic random access memory (around 10 ns) is also not satisfactory [7]. These issues stimulate us to explore novel material system in order to improve the storing media characteristics. Compared with GST, Sb-rich Sb-Te materials have many advantages such as low melting point and fast crystallization [8]. However, it is difficult to guarantee a satisfactory data-retention time at 80°C due to its relatively low crystallization temperature [9]. Recently, the Al-Sb-Te (AST) ternary system has been proposed for application in electric memory [10,11]. Compared with GST, Al-Sb-Te exhibits a high crystallization temperature, good data retention, and high switching speed.

It was reported that merely 0.2% to 1.4% of the total applied energy is effectively used for phase changing, and nearly 60% to 70% of the energy transfers back along the columnar tungsten (W) bottom electrode, having not participated in the heating process of the phase change material (for a T-shaped PCM cell) [12]. Such a low thermal efficiency inevitably leads to a large operating bias/current during the phase change processes. Consequently, one of the effective solutions that has been tried to enhance the thermal efficiency is using an appropriate heating layer between the phase change material layer and the underlying W electrode, or replacing the W plug with some other suitable material. There are some qualified materials that have already been applied in reducing the programming current, such as TiON [13], Ta_2_O_5_[14], SiGe [15], TiO_2_[16,17], SiTaN_*x*_[18], C60 [19], and WO_3_[20]. All these materials have the common physical characteristics of high electrical resistivity and low thermal conductivity. Indeed, a heater material with a large electrical resistivity (>0.1 Ω cm) but low thermal conductivity is most favorable for heat generation and restriction in a PCM cell.

Titanium oxide (TiO_2_) is an n-type semiconductor and has very low thermal conductivity (approximately 0.7 to 1.7 W m^-1^ K^-1^ for 150- to 300-nm thick film) [21]. Note that the thermal conductivity will be even less for a thinner TiO_2_ film. The electrical resistivity of a crystalline TiO_2_ film measured by the van der Pauw method in this study is about 1.2 Ω cm, which is close to the result reported by Xu et al. [17]. In addition, TiO_2_ has a high melting point (approximately 2116 K) and will be thermally stable under high temperature (approximately 900 K) during the reset operation. Generally speaking, with the suitable electrical resistivity, thermal conductivity and thermal stability, a crystalline TiO_2_ layer should hopefully serve as the bottom heating layer in PCM cells to improve the thermal efficiency and, therefore, reduce the power requirement during phase transitions. In this study, the atomic layer deposition (ALD) TiO_2_ was used as a buffer layer which was expected to improve the thermal efficiency and reduce the reset voltage of PCM.

## Methods

**Figure 1:**
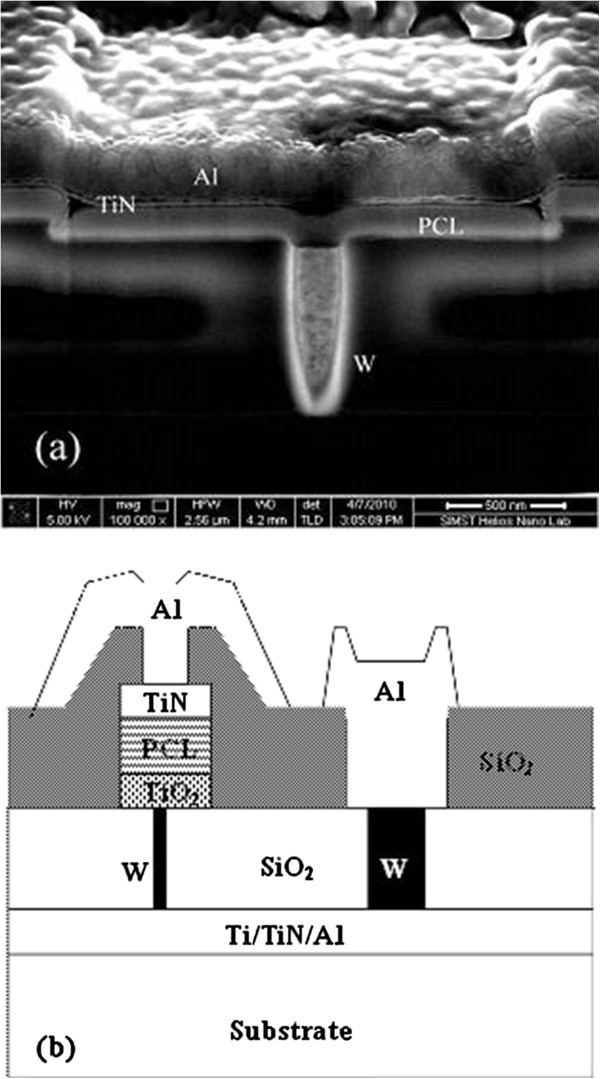
**Cross-sectional structures of PCM cells.** (**a**) Cross-sectional structure of PCM cell without TiO_2_ buffer layer and (**b**) schematic diagram of the cross-section structure of the fabricated cell with TiO_2_ buffer layer.

The PCM cells in this study are fabricated using 0.18 μm CMOS technology. Figure 1a shows a cross-section transmission electron microscopy (TEM) image of the fabricated cell without TiO_2_ buffer layer. The diameter and height of the columnar W electrode are 260 and 700 nm, respectively. Figure 1b shows a schematic diagram of the cross-section structure of the fabricated cell with TiO_2_ buffer layer. The thin TiO_2_ layer was interposed between the phase change layer (PCL) and W plug. A 2-, 4-, and 8-nm thick TiO_2_ buffer layer was deposited by ALD at 400°C using Beneq TFS 500 ALD system (Beneq, Vantaa, Finland). One deposition cycle was composed of Ti precursor (TiCl_4_) pulse (250 ms), 200 sccm N_2_ purge (2 s), water (H_2_O) pulse (250 ms), and 200 sccm N_2_ purge (s2 s). The deposition rate is 0.5 A/cycle. The as-deposited films were crystallized with rutile structure measured by X-ray diffraction. Then, 100-nm thick AST PCL was deposited by magnetron sputtering. The background pressure and Ar gas pressure were 2.0 × 10^-4^ and 0.18 Pa, respectively. The stoichiometry of the deposited films was confirmed by electron dispersive spectroscopy. The Al/Sb/Te ratio was 1:3:1. Then, 20 nm TiN and 200 nm Al were deposited by sputtering as top electrode. For comparison, sputter-deposited AST film without the interposed TiO_2_ layer was also fabricated with the same structure. The electric property tests of PCM were carried out by a Tektronix AWG5012b arbitrary waveform generator (Tektronix, Inc., Shanghai, China) and a Keithley 2602A parameter analyzer (Keithley Instruments, Inc., OH, USA).

## Results and discussion

**Figure 2:**
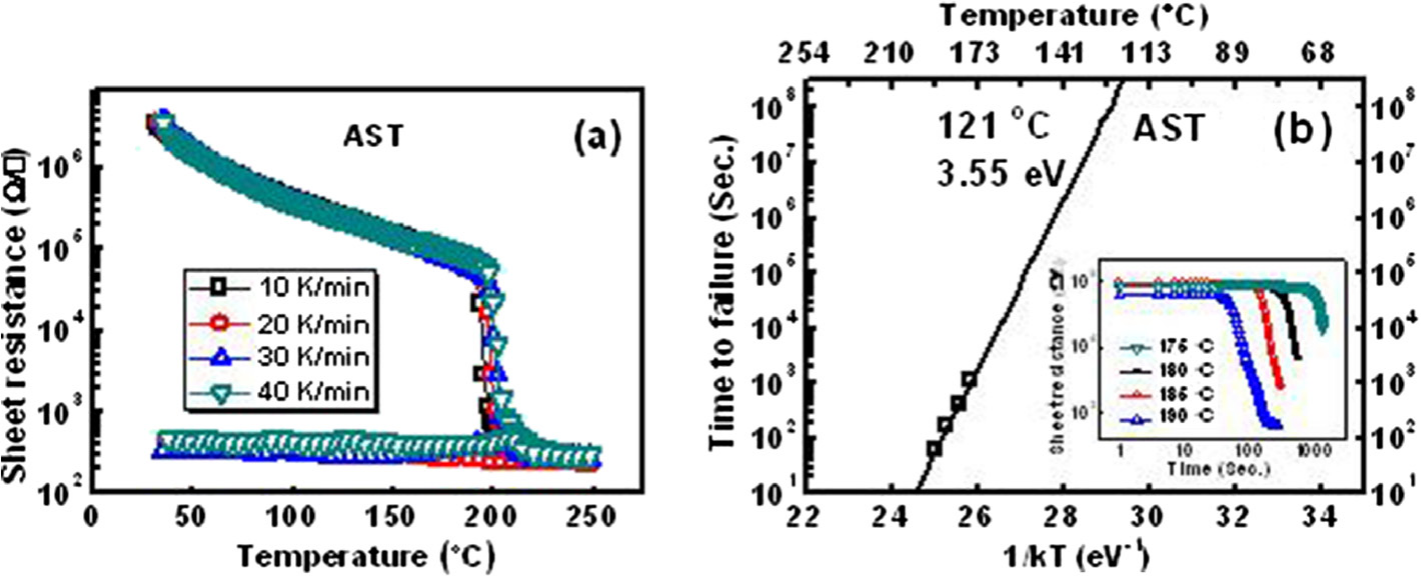
**Sheet resistance change and Kissinger plot.** (**a**) Temperature dependence of the sheet resistance of AST films and (**b**) Kissinger plot from which the *E*_a_ of the amorphous to crystalline transition at *T*_c_ of AST films are determined.

Figure 2a shows the sheet resistance change of AST films as a function of temperature. The sample with a thickness of 100 nm was prepared on the SiO_2_/Si(100) by sputtering at room temperature. Upon heating, the sheet resistance of AST films decreased with a rapid drop at the crystallization temperature (*T*_c_). The *T*_c_, defined by the temperature corresponding to minimum of the first derivative of *RT* curve, was about 198°C which is higher than that of GST films (approximately 145°C) [22]. To check the crystallization kinetics, electrical resistivity was *in situ* measured with increasing temperature with various heating rates *dT*/*dt*. Applying Kissinger’s analysis which relates the transition temperature *T*_c_, the rate of heating (*dT/dt*), and the activation energy (*E*_a_) for crystallization by the formula below:

(1) $$\text{ln}\left[\left(\text{dT}/\text{dT}\right)/{T_{c}}^{2}\right]=C-\left(E_{a}/k_{B}T_{c}\right)\text{,}$$

where *C* is a constant, *k*_B_ is the Boltzmann constant, a plot of ln[(*dT/dt*)/*T*_c_^2^ against 1/*T*_c_ yields a straight line with slope, -*E*_a_/*k*_B_. From the Kissinger plot shown in Figure 2b, the activation energy for crystallization of AST was determined to be about 3.55 eV which is higher than that of GST films (approximately 2.01 eV) [22]. It has to be noted that the high crystallization temperature and high activation energy of AST offer a large benefit for a stable operation of the PCM device because the cells in the amorphous state tend to switch to the crystalline state due to cross talk, i.e., the heat dissipation from other cells.

**Figure 3:**
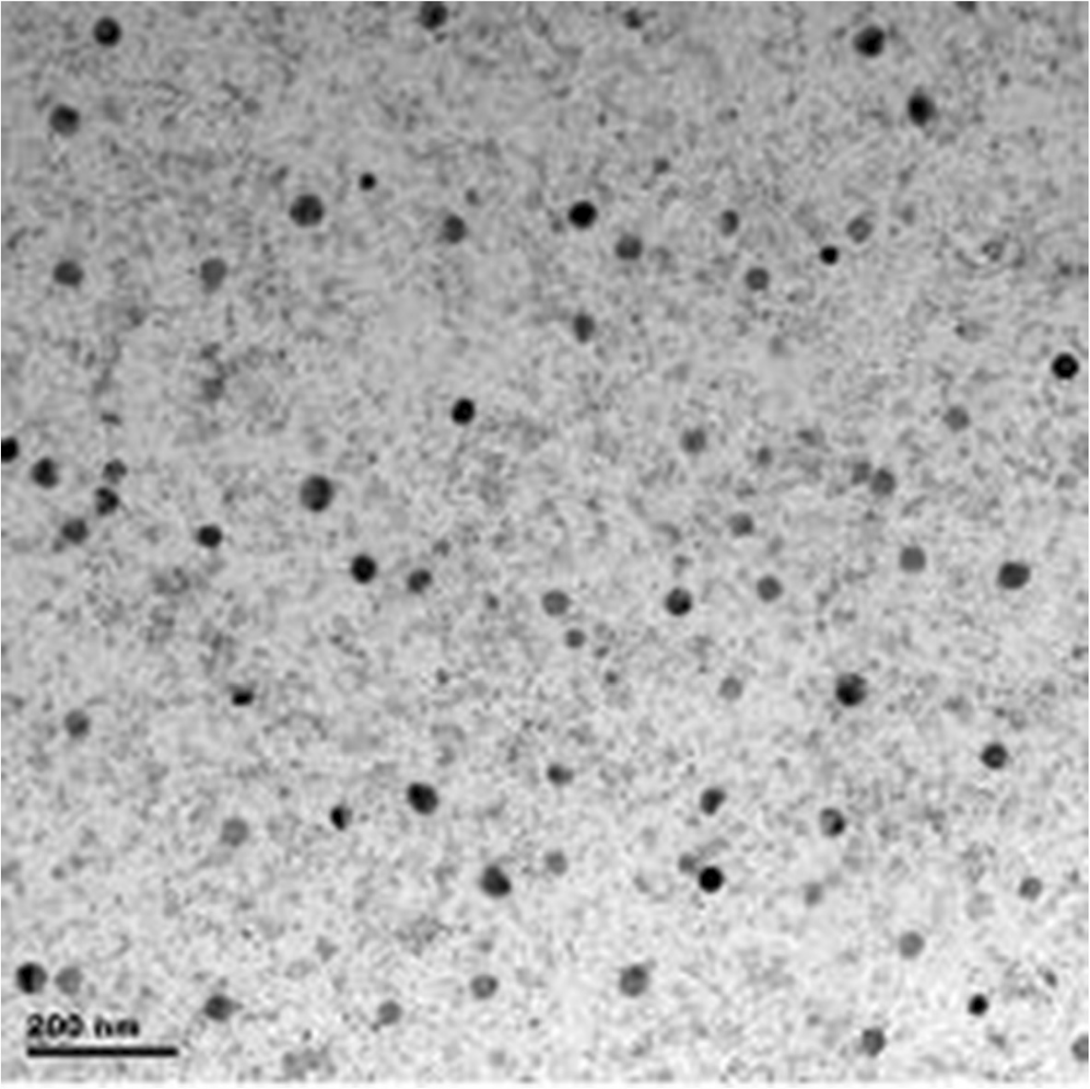
TEM image of AST film after a 2-min heating at 400°C.

The bright-field TEM was used to study the structure of thin films. Figure 3 shows the TEM image of AST film after a 2-min heating at 400°C in Ar atmosphere; nanocrystals (dark spots) were observed. Peng et al. reported that an embedded crystal structure of hexagonal (Sb_2_Te) and monoclinic (Al_2_Te_3_) phases can be found in AST materials [10]. The black area in the image results from an overlap of Sb_2_Te and Al_2_Te_3_ crystalline grains. The overlap of grains will lead to a larger local density, and the incident electrons will be more scattered by these areas.

**Figure 4:**
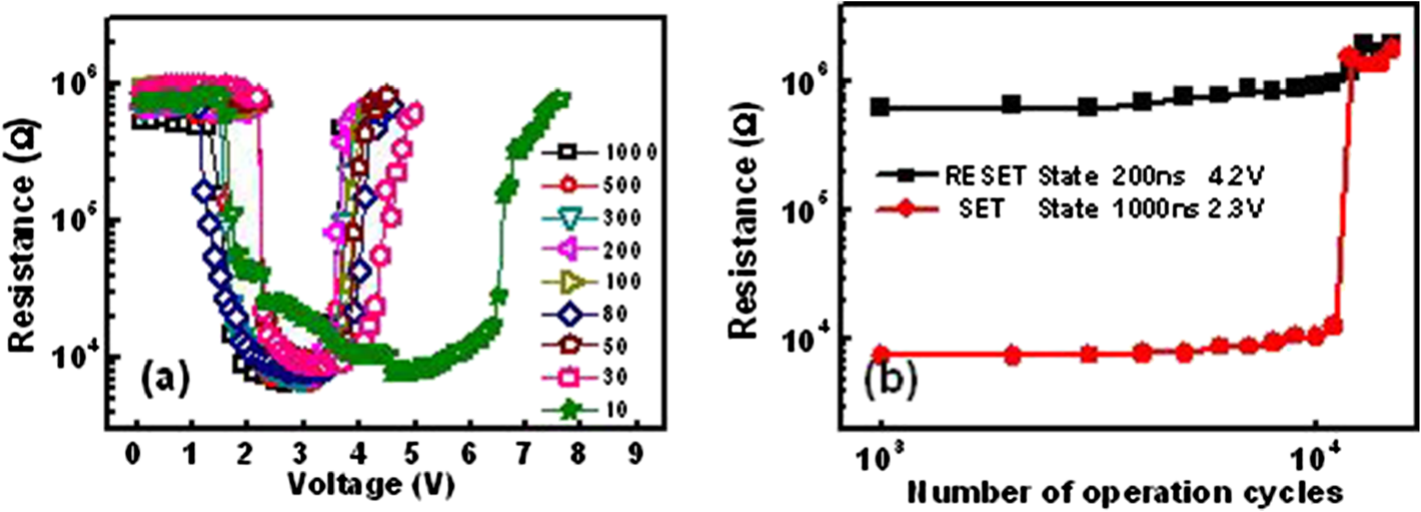
**Resistance voltage and endurance characteristics.** (**a**) Resistance voltage characteristics of PCM cell with AST films by different voltage pulse widths. (**b**) Endurance characteristics of the PCM cell with AST film.

The phase transition of PCM cell can be characterized from the relation between the cell resistance and the corresponding amplitude of voltage pulse or current pulse (so called *R*-*V* or *R*-*I* curve). The measured *R*-*V* curves for AST PCM cells with different pulse width are shown in Figure 4a. Reversible phase-change process has been observed. As revealed, once the programming voltage increases beyond the threshold voltage, the cell resistance starts to drop due to the crystallization of AST alloy and then reaches a minimum, which is corresponding to the set resistance. When the voltage is further increased, the resistance again rises and then returns to the reset state. It is clear that the set resistance decreases with the pulse width. The higher set resistance resulted from a shorter pulse implies that incomplete crystallization states are formed after set programming. It can be seen from Figure 4a the resistance of the AST devices dramatically increased by two orders of magnitude at a reset voltage of around 4.1 V (at 50 ns).

**Figure 5:**
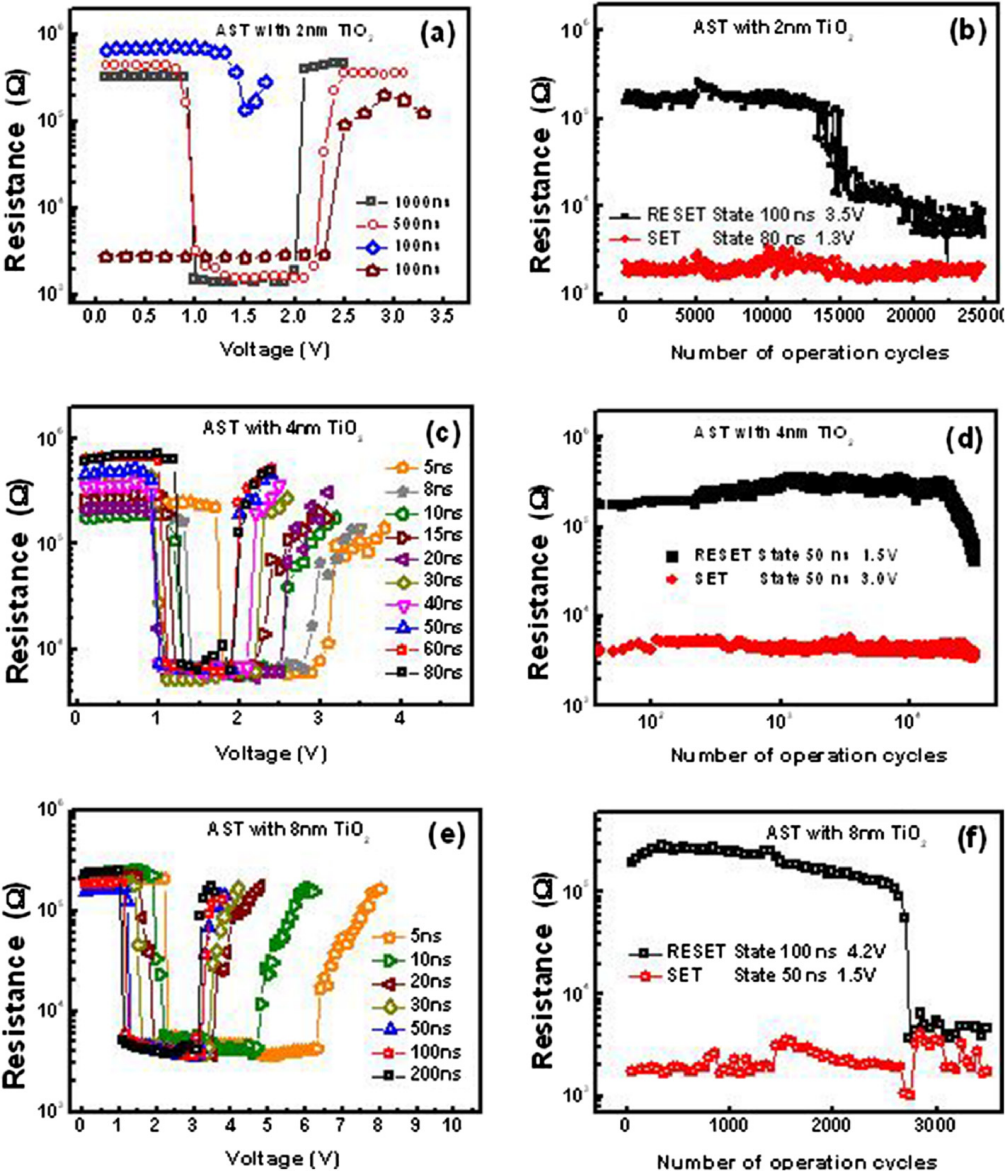
**Resistance voltage characteristics of PCM cell at different pulse widths.** (**a**) 2, (**c**) 4, and (**e**) 8 nm TiO_2_. Endurance characteristics of the PCM cell (**b**) with 2, (**d**) 4, and (**f**) 8 nm TiO_2_.

Figure 5a,c,e shows the variations in cell resistance with the 2-, 4-, and 8-nm thick TiO_2_ buffer layer as a function of the voltage for the set and reset operations, respectively. For the device with 2 nm TiO_2_, as shown in Figure 5a, a 100-ns width pulse fails to set the cell and a pulse width of 100 ns is insufficient for a complete reset programming, suggesting that 2 nm TiO_2_ layer indeed leads to a slower crystallization process, thus longer write time for the set operation. For a device with 8 nm TiO_2_, as shown in Figure 5e, a 5-ns pulse can trigger reversible phase-change of the cell, and the reset voltage of approximately 3.8 V (at 50 ns) of the cell is clearly lower than that of the AST cells (about 4.1 V) without TiO_2_ layer. With 50-ns, pulse reset voltage of 2.4 V was achieved for the device with 4 nmTiO_2_ layer (in Figure 5c), which is only about half of the voltage required by the device without TiO_2_ buffer layer. The voltage reduction could be understood from the high Joule heating efficiency and the good thermal confinement. The oxide interfacial layer prevents heat generated in the programming volume of the AST from diffusing to the plug, which has high thermal conductivity, resulting in low power set/reset operation. Similar improvement has been reported on other kinds of oxide interfacial heater layers [23,24]. Besides that, both of the resistances in amorphous and crystalline states retained at the same levels after inserting the TiO_2_ layer. These results prove a fact that the inserted TiO_2_ layer will not drift the resistance but can sharply diminish the operation voltage, which will be helpful to solve the difficult problem in the compatibility with the continuing scaling down dimension in CMOS process. It is worthy to point out that the set resistance is very stable for the cells with TiO_2_ layer at different pulse widths, suggesting that the TiO_2_ layer helps to raise the temperature profile within the phase change film and, thereby, enhances the heat-induced phase transition process. Furthermore, there are some other advantages of TiO_2_ such as easily fabricated, no pollution, fully compatible with CMOS process, and avoids the diffusion between phase change material and bottom electrode.

Figure 4b and Figure 5b,d,e show the repeatable resistance switching between the set and reset states of the cells without and with TiO_2_ layer, respectively. For the device without TiO_2_, as shown in Figure 4b, the endurance capability keeps about 20,000 cycles before the presence of resistance disorder with a set stuck failure mechanism. For the device with 2 nm TiO_2_, as shown in Figure 5b, the reset resistance reduced gradually during the cycling. After 14,000 cycles, the reset resistance dropped rapidly, leading to the endurance failure by losing the set and reset resistance window. For the device with 8 nm TiO_2_, as shown in Figure 5f, the endurance capability keeps about 2,700 cycles before the presence of resistance disorder with a reset stuck failure mechanism. Good endurance characteristics (>10^4^ cycles) was found in the cell with 4-nm TiO_2_ buffer layer. The low resistance state maintained around 10^3^ Ω magnitude, and the high resistance state kept on 10^5^ Ω level, indicating a satisfactory data resolution capability for random access memory application. The difference cyclic operation behavior shown in Figure 4b and Figure 5b,d,e suggested the different performance degradation processes for the device with and without TiO_2_ layer, which is currently under investigation. Among the various thicknesses of the TiO_2_ buffer layer, 4 nm was the most appropriate thickness that maximized the improvement with negligible sacrifice of the other device performances, such as the reset/set resistance ratio, voltage window, and endurance.

## Conclusions

This paper reports an efficient method for reducing the reset voltage and power of the conventional T-shaped PCRAM, which has the potential to replace the current nonvolatile memories. We inserted TiO_2_ layer between phase change memory and bottom electrode to increase the utilization of the Joule heat and reduce the heat dissipation. Due to the suitable electrical resistivity and the low thermal conductivity of TiO_2_ film, the overall set resistance of the PCM cell will not be greatly increased, while the remarkably increased overall thermal resistance helps to reduce the reset voltage.

## Competing interest

The authors declare that they have no competing interests.

## Authors’ contributions

SS and ZS conceived the study and revised the manuscript. CP and LG carried out the XRD and TEM characterizations. YG and ZZ participated in the sample preparation. YL and DY participated in the fabrication of the device. LW and BL read the manuscript and contributed to its improvement. All the authors discussed the results and contributed to the final version of the manuscript. All the authors read and approved the final manuscript.

## Authors’ information

SS is an associate professor at the State Key Laboratory of Functional Materials for Informatics, Shanghai Institute of Micro-system and Information Technology, Chinese Academy of Sciences.
